# Bibliometric-based visualization analysis of hotspots and trends in falls research among older hospitalized patients (2013–2023)

**DOI:** 10.3389/fmed.2024.1433193

**Published:** 2025-03-03

**Authors:** Yang Dong, Dandan Liu, Ya Yu, Ziyu Xiong, Hongying Rao

**Affiliations:** ^1^Department of Geriatrics, Guangzhou First People’s Hospital/the Second Affiliated Hospital, School of Medicine, South China University of Technology, Guangzhou, China; ^2^Department of Thoracic Surgery, State Key Laboratory of Oncology in South China, Collaborative Innovation Center for Cancer Medicine, Sun Yat-sen University Cancer Center, Guangzhou, China

**Keywords:** older hospitalized patients, fall, research hotspots, bibliometrics, visualization analysis

## Abstract

**Purpose:**

We utilized Citespace 6.2 R4 software to visualize and analyze the literature published within the past decade (2013–2023) pertaining to falls in elderly hospitalized patients, with the objective of examining the progression and patterns of focal points within this research domain. Our aim is to offer a valuable reference and foundation for related studies and to provide guidance for healthcare professionals and researchers in advancing further exploration and implementation of strategies for preventing and managing falls in elderly patients.

**Methods:**

We conducted a literature search in the Web of Science database using keywords such as “older hospitalized patients” and “fall” to develop a search strategy that was highly relevant to the topic of falls among older hospitalized patients. We further limited the literature time range to January 1, 2013, to December 31, 2023, to capture the latest research trends over the past decade. In terms of literature type, we focused on “article” and excluded conference papers, reviews, editorials, etc., to ensure the scientific rigor and reliability of the study. During the screening process, we excluded duplicate publications and those documents that were not directly related to falls among older hospitalized patients, such as those primarily discussing falls in other age groups or non-hospital environments. Although our search had no language restrictions, we only included English-language literature to ensure consistency and readability of the language. Additionally, we evaluated the quality of the literature and excluded those with unclear research methods or unreliable results to ensure the reliability of the research findings. Subsequently, we utilized CiteSpace 6.2 R4 software to generate a knowledge map for visualization and analysis.

**Results:**

Our analysis included a total of 321 articles. The results showed that the majority of contributions in this field came from the United States and China, both of which exhibited an intermediary centrality >0.1, indicating their significant influence. Harvard University became the leading institution with the most published articles (*n* = 8), while Imagama was identified as the most prolific author (*n* = 6). Moving forward, combining keywords with the strongest citation bursts, it is expected that the research trends in this field in the future will focus on epidemiology, aging, and health-related topics.

**Conclusion:**

Our study presents a comprehensive investigation into the evolution and trends of research focal points regarding falls in elderly hospitalized patients from 2013 to 2023. Our findings reveal a significant increase in attention toward this research area over the past decade, with a growing number of studies being conducted. Fall risk assessment, prevention strategies, rehabilitation interventions, and costs associated with falls have emerged as the primary research focal points within this field. Furthermore, epidemiology, aging population dynamics, and health outcomes continue to be enduring areas of interest for researchers post-2018 and warrant additional emphasis from scholars.

## Introduction

Population aging is a pressing issue that necessitates attention in the advancement of human society, and it is an inevitable consequence of demographic evolution, which exhibits a trend toward aging in China. With the decrease in fertility rates and advancements in medical standards, there has been a steady increase in the number of individuals aged 60 and above within the Chinese population. According to China’s seventh demographic statistics, more than 20% of the population now falls into this age group, indicating a worsening trend toward an aging national society (1).

The term “fall” is commonly used to describe an involuntary descent or collapse of an individual while in a standing or walking position, typically resulting from a loss of equilibrium or other contributing factors (2). According to the International Classification of Diseases, 11th Revision (ICDE-11) falls include the following two categories: (i) falls from one plane to another and (ii) falls on the same plane (3). The prevalence of falls and the rates of morbidity and mortality both exhibit an upwards trend with advancing age. In China, the prevalence of falls among older patients hospitalized over 65 years old reaches 30%, while it increases to 50% among those aged over 80 years (4). The occurrence of falls and their associated injuries presents a significant obstacle to the process of normal aging, while also imposing a substantial financial strain on families, communities, and governmental resources (5). According to relevant research, the average increase in hospitalization expenses for patients who experience falls is estimated at US$6,669 per person, while the costs associated with fall-related injuries range from US$12,000 to US$23,000 per individual (6). Falls pose a significant health risk to older people, and there is an urgent need for the development of effective global fall prevention strategies. Consequently, numerous scholarly studies focusing on falls in the older population have been conducted worldwide.

We conducted a comprehensive review of articles from the WOS core database over the past decade and utilized CiteSpace 6.2 R4 software to visualize and analyze literature information, aiming to present hotspots and trends in research on falls among hospitalized older patients in a more intuitive manner. This analysis is intended to provide valuable guidance and a reference for future studies in this field.

## Methods

### Data sources and selection

The Web of Science, a research database developed by Clarivate Analytics, offers extensive access to academic journals, conference proceedings, and other scientific literature. It is widely utilized by researchers, academics, and students for locating scholarly articles, tracking citations, and identifying research trends.

In this study, we conducted a comprehensive review of the literature encompassed in the WOS core database utilizing the following search strategy: [TS = (“older hospitalized patients” OR “elderly hospitalized patients” OR “older inpatients”) AND TS = (“fall” OR “falls” OR “stumble” OR “tumble” OR “fall related injuries”)], we further limited the literature time range to January 1, 2013, to December 31, 2023, to capture the latest research trends over the past decade. In terms of literature type, we focused on “article” and excluded conference papers, reviews, editorials, etc., to ensure the scientific rigor and reliability of the study.

### Article exclusion criteria

During the screening process, we excluded duplicate publications and those documents that were not directly related to falls among older hospitalized patients, such as those primarily discussing falls in other age groups or non-hospital environments. Although our search had no language restrictions, we only included English-language literature to ensure consistency and readability of the language. Additionally, we evaluated the quality of the literature and excluded those with unclear research methods or unreliable results to ensure the reliability of the research findings.

### Data analysis method

CiteSpace, a bibliometric analysis software developed by Dr. Chaomei Chen of Drexel University, is utilized for the purpose of scientific knowledge mapping and visualization analysis (7). The software is capable of analyzing literature citation information, author collaboration, and current research topics. It can also generate visual maps using graph theory and bioinformatics to assist researchers in identifying research hotspots and disciplinary trends within the field (8). The utilization of CiteSpace can assist researchers in gaining insights into significant research trends within the field, academic partnerships, and collaborations among scientists, thereby optimizing the strategy and direction of scientific inquiry. In this study, we utilized CiteSpace 6.2 R4 software to conduct bibliometric-based Visualization Analysis. To ensure the transparency and reliability of the analysis, we configured the software parameters as follows.

Before data import, we conducted thorough data cleaning, including removing duplicate literature records and those that are not related to the research topic. The literature was imported into plain text software, which contained all records, to construct a comprehensive database. Subsequently, the included literature was visualized and analyzed, with node types selected as country, institution, author, and keywords for drawing the visual knowledge graph. The size of the nodes in the knowledge graph generated by the software reflects the number of articles published by research authors/institutions/countries in the field. Furthermore, links between nodes represent cooperative relationships among research authors/institutions/countries, with link thickness indicating their strength.

The links were set to Cosine, the scope was set to Within Slices, and the time slice was set to 1 year. The TOP N was set to 50 to filter out literature with a higher citation rate or occurrence rate per year, while the *g*-index was set to 25. Pruning was chosen as Pathfinder for sliced networks, and all other settings were left at their default values, the above settings remove unimportant nodes and links, ensuring clarity and relevance in the visualization results. During the clustering analysis performed by the software, Modularity Q represented the clustering structure, and Weighted Mean Silhouette represented the degree of reasonableness of the clusters. A *Q* value greater than 0.3 indicated a clear and significant clustering structure, while an *S* value greater than 0.5 indicated strong cluster reasonableness (9).

## Results

### Overview of the results

Our study included 321 articles, within which the United States and China stood out as major contributing countries to the field of research, with Harvard University and scholar Imagama being notable for their significant contributions. The co-occurrence of keywords and keywords cluster covered various aspects of fall research among elderly patients, including diseases (such as Parkinson’s disease), technological applications (such as machine learning), characteristics of the elderly population, medical interventions (such as prescriptions), psychological factors related to falls (such as fear of falling), and economic burdens. The steady increase in annual number of publications reflects a continuing upward trend in the development of this field of research.

### Annual number of publications

**Figure 1:**
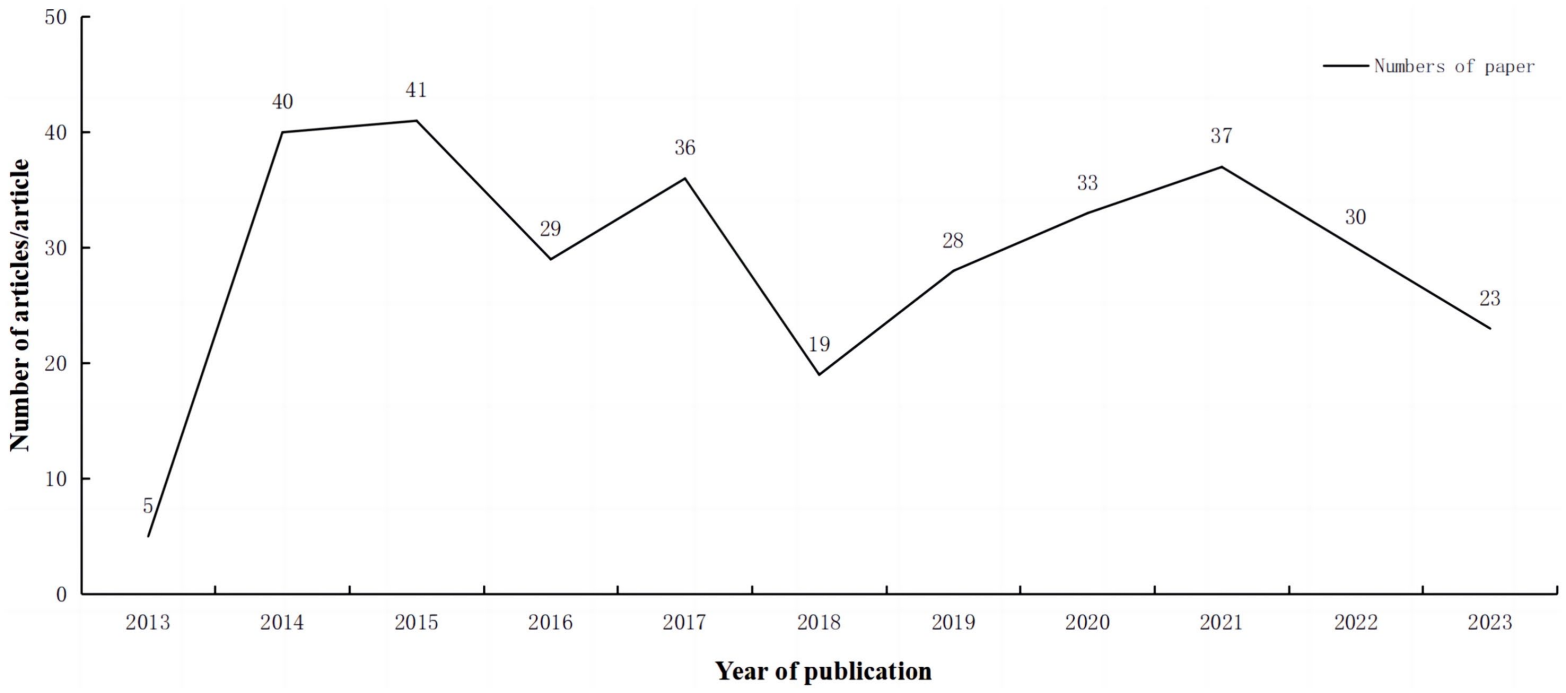
Annual number of publications.

We conducted a comprehensive search of recent literature in the WOS core database, encompassing the time period from January 1, 2013, to November 1, 2023. A total of 435 documents were retrieved and subsequently screened, resulting in the inclusion of 321 articles. The number of articles published in 2013 was limited to five; however, there has been a discernible increase in scholarly interest within this field over time, with an annual average publication rate of 32 articles during this 10-year period. Additionally, there was a notable upwards trend in the number of articles published over the course of this decade, as depicted in Figure 1.

### Distribution of published journals

The top 10 journals published in studies of falls in hospitalized older patients are shown in Table 1. BMC GERIATRICS (*n* = 41) was the journal that published the most research in this field, and the IF = 4.1 of both the Journal of the American Geriatrics Society and BMC GERIATRICS were the two journals with the highest impact factors among these 10 journals. These 10 journals cover fields such as Geriatrics & Gerontology, Multidisciplinary Science, General Internal Medicine, Emergency Medicine, and Endocrinology Metabolism, and so on.

**Table 1:** Top 10 journals for the publication of studies on falls in hospitalized older patients.

No.	Title of journal	Count	IF	Country	Research area
1	BMC Geriatrics	41	4.1	England	Geriatrics & Gerontology
2	PLoS One	31	3.7	United States	MULTIDISCIPLINARY SCIENCE
3	Aging Clinical and Experimental Research	21	4	Italy	Geriatrics & Gerontology
4	Clinical Interventions in Aging	21	3.6	New Zealand	Geriatrics & Gerontology
5	Cureus Journal of Medical Science	20	1.2	United States	GENERAL INTERNAL MEDICINE
6	Injury International Journal of the Care of the Injured	18	2.5	England	EMERGENCY MEDICINE
7	Osteoporosis International	17	4	England	ENDOCRINOLOGY METABOLISM
8	Geriatrics Gerontology International	13	3.3	Japan	Geriatrics & Gerontology
9	Journal of the American Geriatrics Society	13	4.1	United States	Geriatrics & Gerontology
10	BMJ Open	12	2.9	England	GENERAL INTERNAL MEDICINE

### Co-occurrence analysis of countries/regions and institutions

Table 2 presents the top 10 countries/regions and institutions involved in research on falls among hospitalized older patients. The United States (*n* = 55), China (*n* = 42), and Japan (*n* = 32) are the three leading countries in terms of publication quantity, while Harvard University (*n* = 8), Harvard Medical School (*n* = 7), and the University of Sydney (*n* = 7) are among the top institutions with the greatest number of publications, primarily representing higher education institutions.

**Table 2:** Top 10 countries/regions and institutions participating in studies of falls in older hospitalized patients.

No.	Countries/Regions	Count	Institutions	Count
1	United States	55	Harvard University	8
2	Peoples R China	42	Harvard Medical School	7
3	Japan	32	University of Sydney	7
4	Spain	20	Nagoya University	6
5	Australia	20	Brigham & Women’s Hospital	5
6	Turkey	16	Capital Medical University	5
7	Italy	16	Aalborg University Hospital	4
8	Canada	15	Aalborg University	4
9	France	14	University of Bern	4
10	England	11	Geriatric Research Education & Clinical Center	4

**Figure 2:**
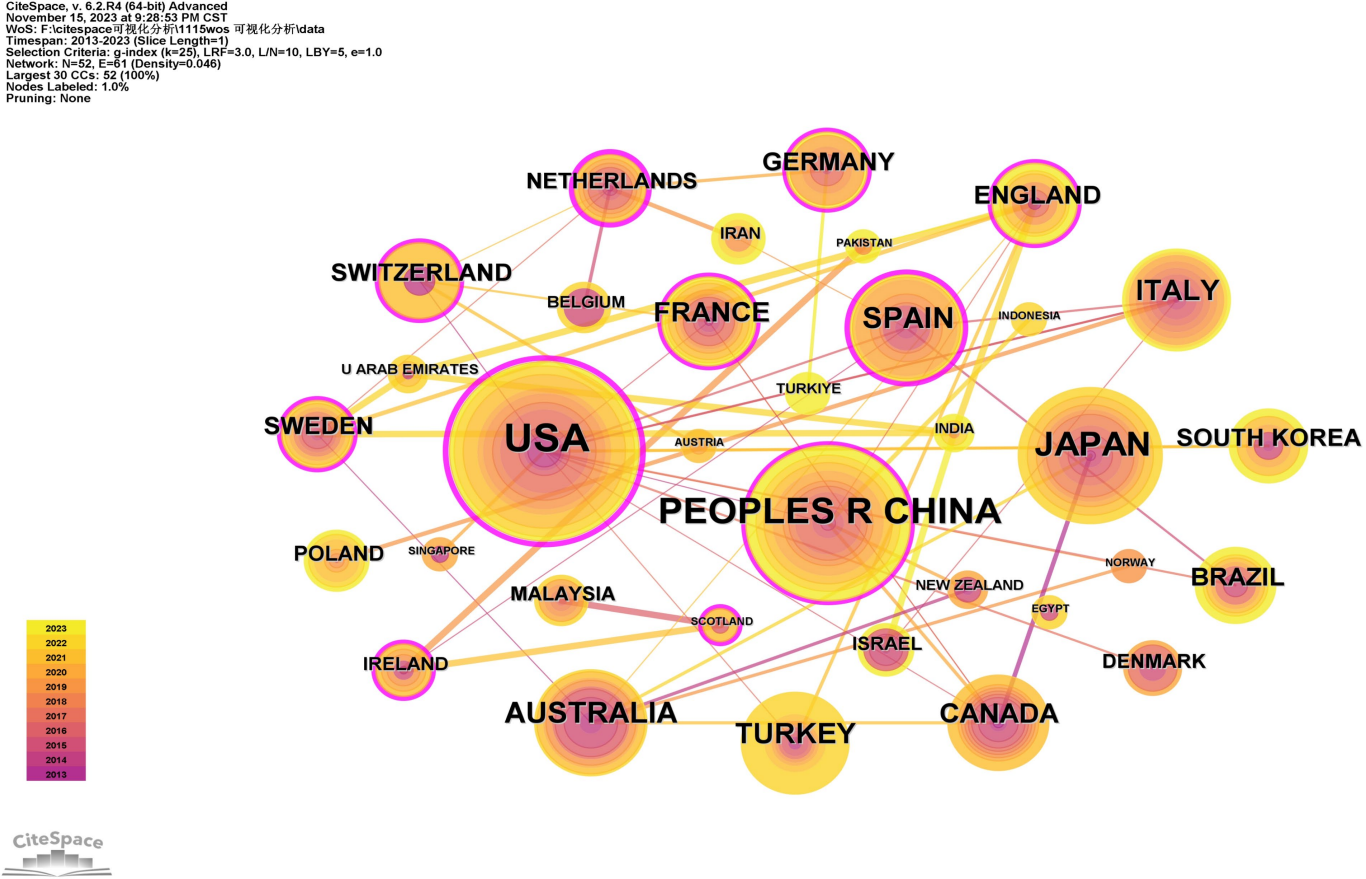
Visualization of country/region co-occurrence.

The co-occurrence visualization of countries/regions is depicted in Figure 2, revealing that a total of 52 countries have engaged in relevant research on falls in older hospitalized patients over the past decade. The United States exhibits a mediational centrality of 0.51, surpassing the threshold of 0.1 and positioning itself at the core of this research field with significant influence. Furthermore, the dense and prominent links between the United States and Canada, the United Kingdom, Australia, and other Western countries indicate strong collaborative relationships. Conversely, while China also demonstrates a mediational centrality >0.1, its cooperation with other countries is limited primarily to Asian nations. Therefore, fostering collaboration with Western countries may yield more extensive research outcomes and academic contributions in this field.

**Figure 3:**
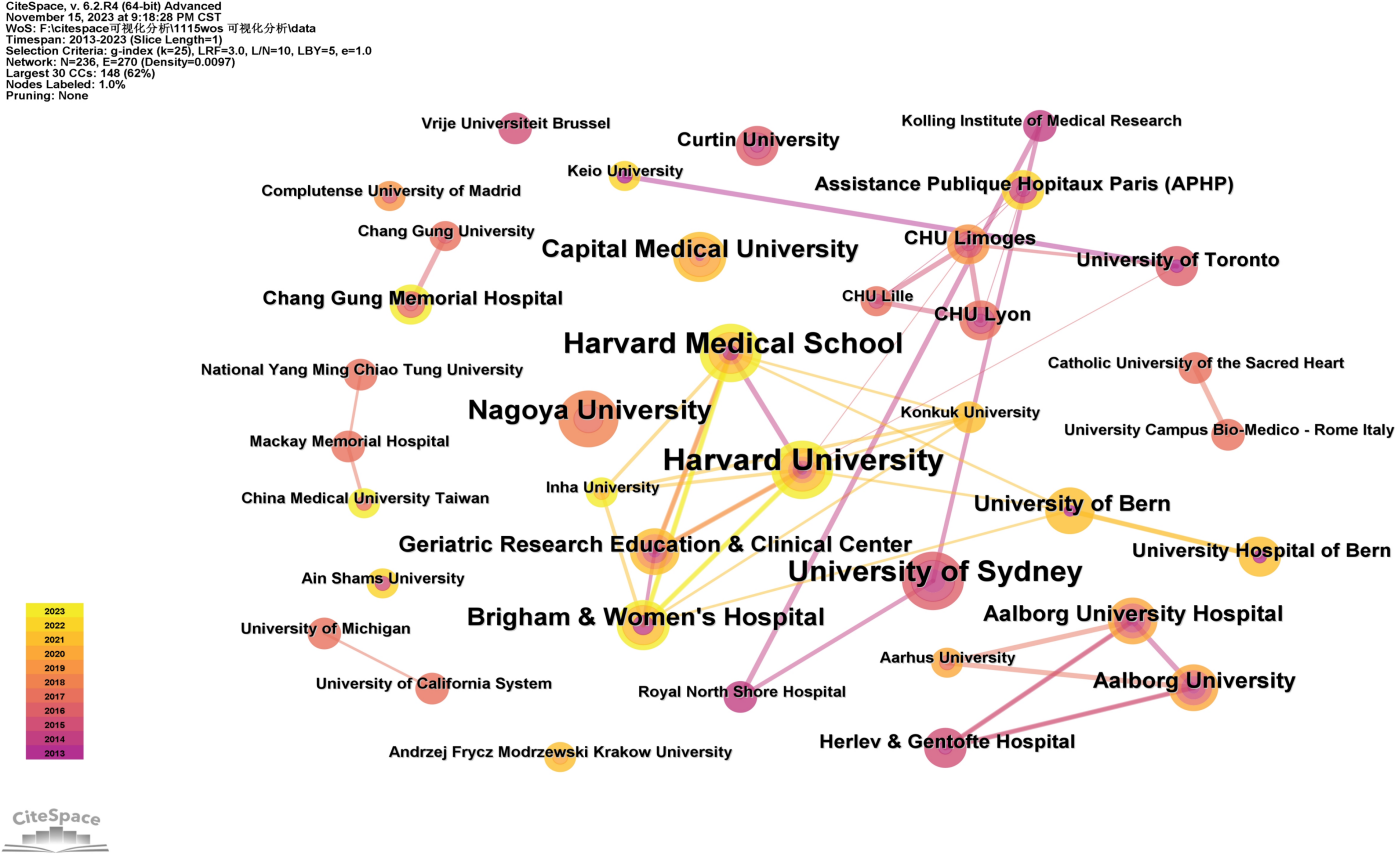
Visualization of institutional co-occurrence.

As shown in Figure 3, a total of 236 institutions have conducted research in the field of falls in older hospitalized patients in the past decade, and most of them are institutions of higher education, among which Harvard University, Harvard Medical School, the University of Sydney, the University of Nagoya, and Capital Medical University are the top five institutions in terms of the number of publications with a total of 33 articles, and Harvard University, Harvard Medical School, Brigham & Women’s Hospital, and the University of Toronto are the top institutions in terms of the number of publications with a high intensity of collaboration. In addition, Harvard University, Harvard Medical School, Brigham & Women’s Hospital, and the University of Toronto collaborated closely and intensively with each other, with Harvard University (*n* = 8) being the top institution in terms of the number of articles published in this field.

### Co-occurrence analysis of authors

**Figure 4:**
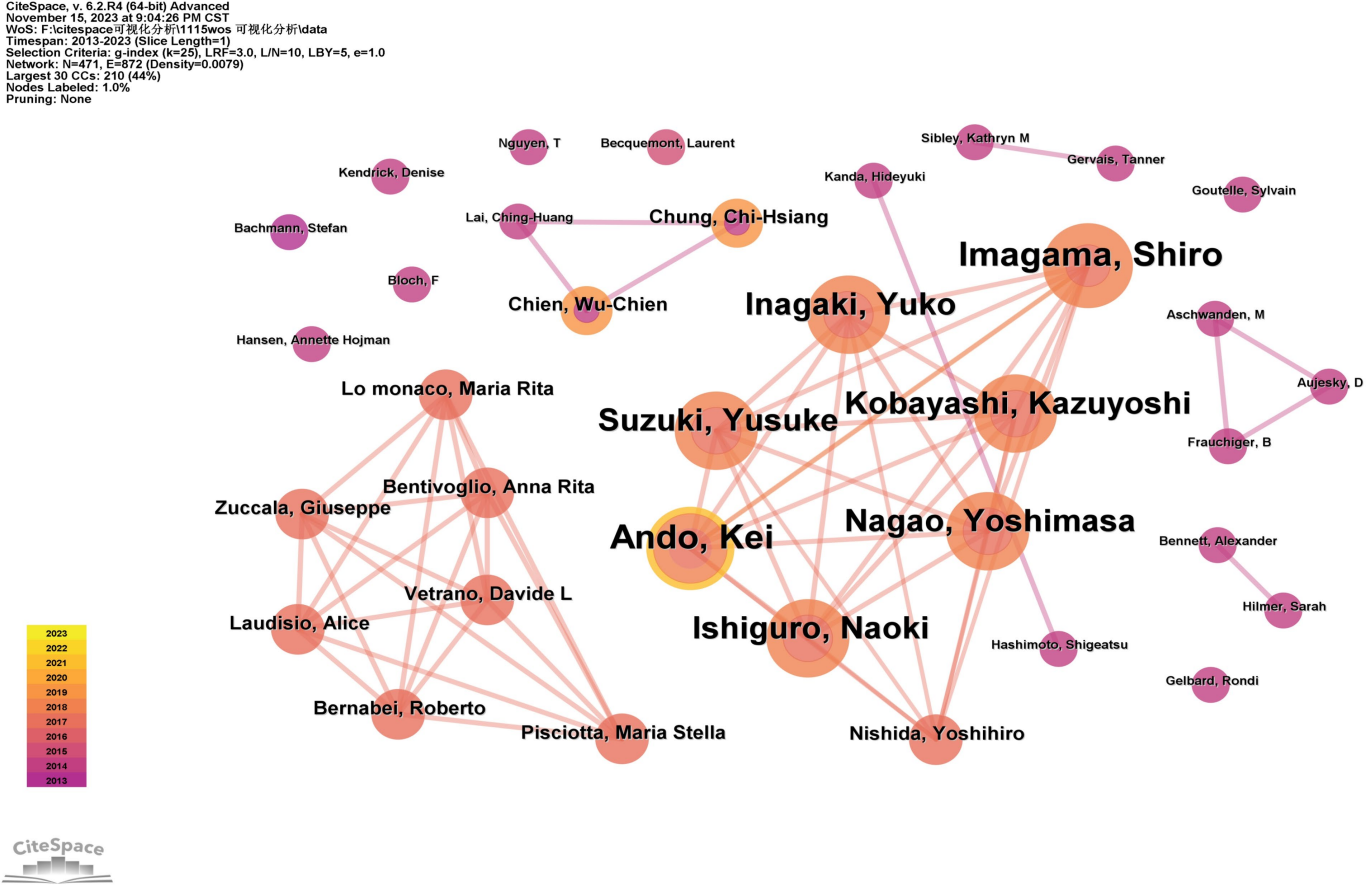
Author co-occurrence visualization.

The visualization of authors’ co-occurrence is shown in Figure 4, in which Imagama (*n* = 6) and Ando (*n* = 6) are the authors with the most publications in this field in the last 10 years, and the visualization shows that the cooperation between authors is relatively limited, and only some of the main researchers collaborate in the network; for example, Imagama, Ando, and Inagaki collaborate in close cooperation with high intensity, and Bernabei, Bentivoglio, and Maria also form the main researcher collaborative network.

### Co-occurrence analysis of keywords

The article keywords typically encompass the terminology utilized to delineate the subject matter, substance, or objective of a literary, scientific, technical, or scholarly manuscript. Normally, literature keywords are ascertained through an analysis and synthesis of the literature’s content (10). In CiteSpace, keyword co-occurrence analysis can be utilized to demonstrate the frequency and pattern of simultaneous appearances of a single keyword or a cluster of keywords in scholarly literature. This type of analysis is instrumental for researchers in identifying research trends, interconnected topics, and forefront issues within their field of study.

**Figure 5:**
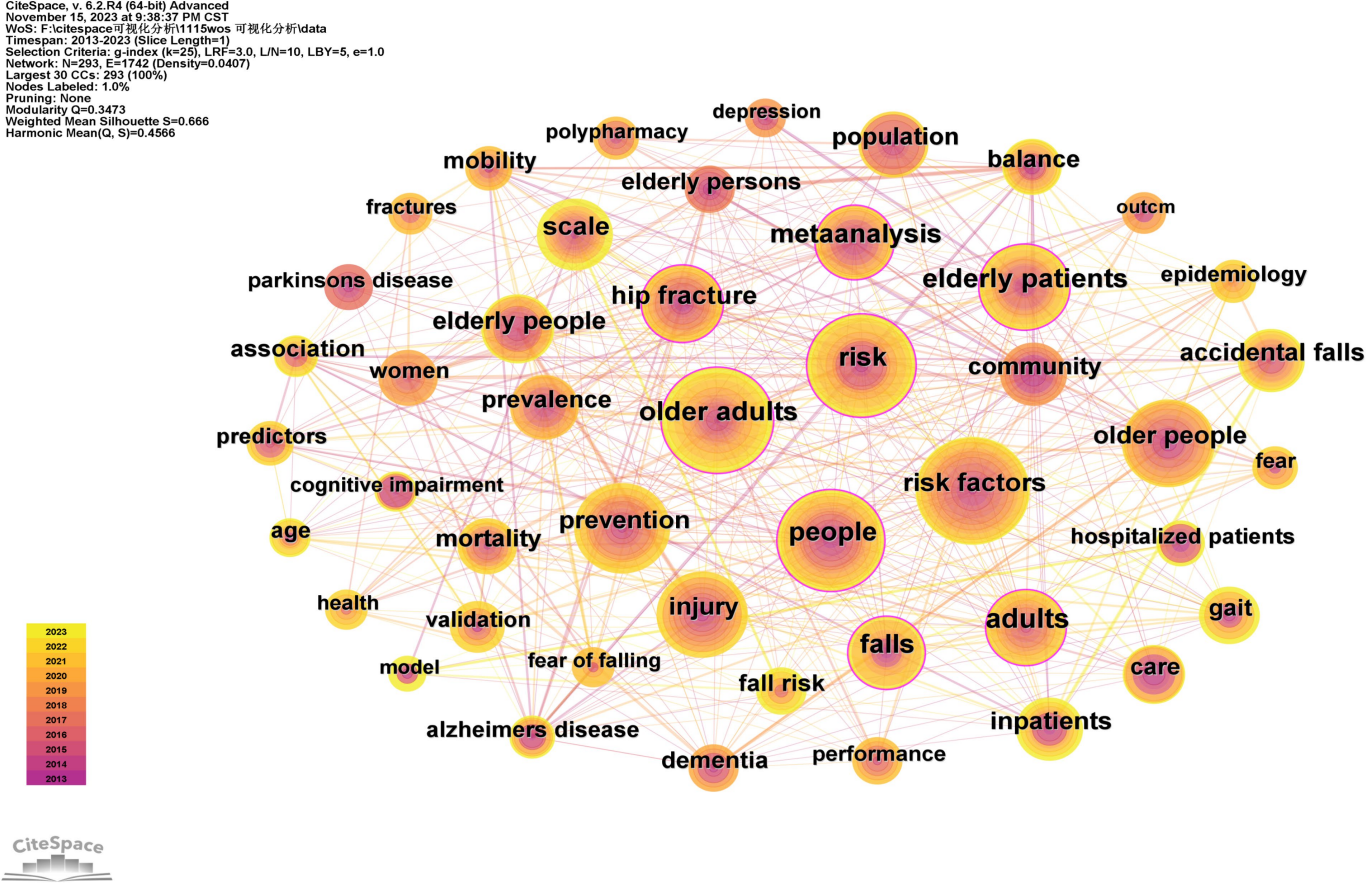
Keyword co-occurrence visualization.

The co-occurrence visualization of keywords is depicted in Figure 5, with core keywords exhibiting centrality >0.1 including older adults, risk factors, older patients, meta-analysis, hip fracture, and falls. The frequency of these keywords exceeded 50, indicating their significance as research hotspots in the field of fall research over the past decade. The substantial volume of studies underscores the prominence of this topic within the realm of falls among older hospitalized patients.

### Analysis of keyword cluster

**Figure 6:**
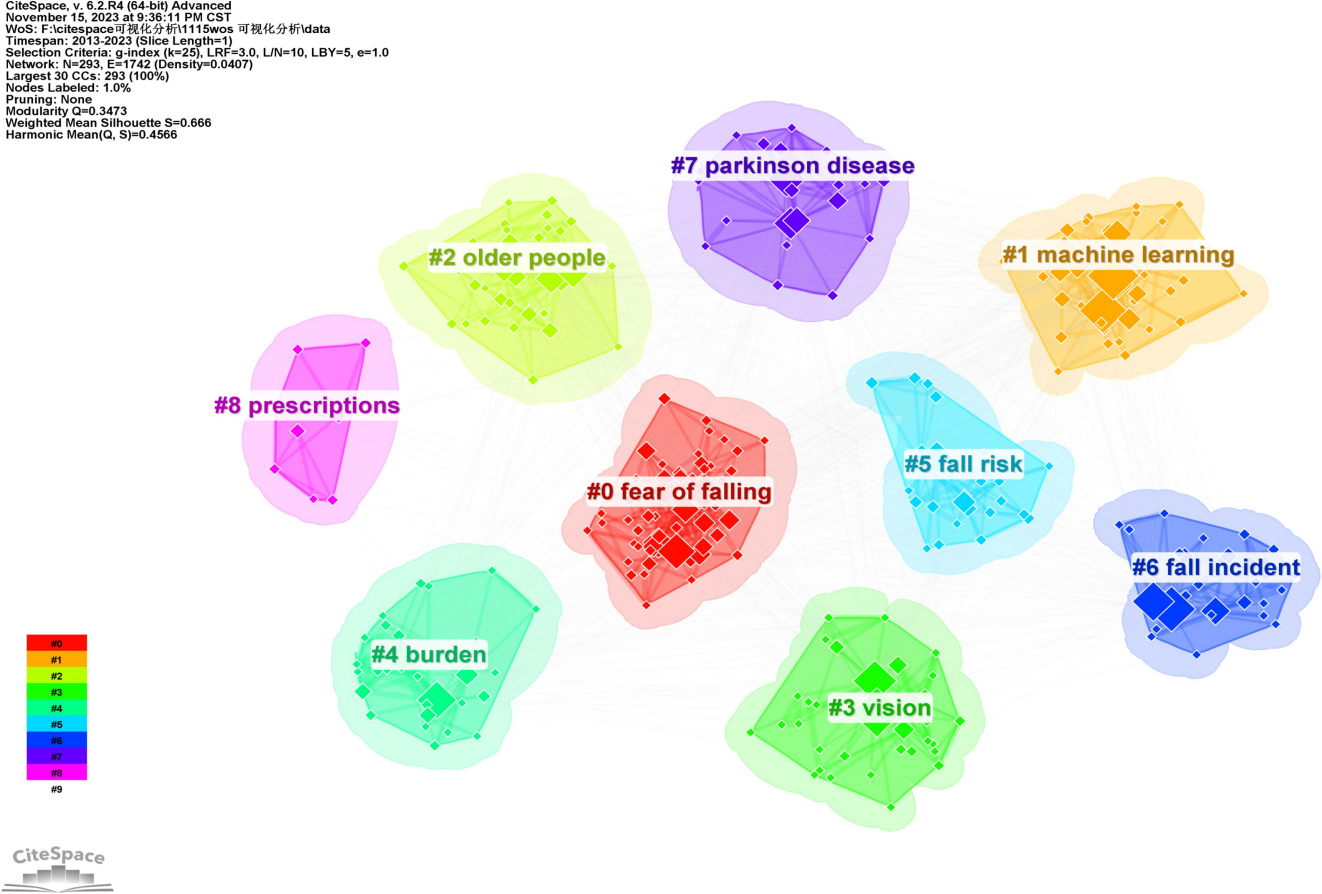
Keyword cluster visualization.

We utilized CiteSpace 6.2 R4 software to employ the logarithmic likelihood ratio (LLR) method for clustering keywords based on the aforementioned procedure. The visualization of keyword clusters is presented in Figure 6, demonstrating a *Q* value of 0.3473 > 0.3 and an *S* value of 0.666 > 0.5. These results indicate a clear and significant structure in the keyword clustering, with a strong degree of rationality.

The number of cluster labels exhibits a negative correlation with the quantity and significance of keywords encompassed within the clusters, such that smaller cluster labels are associated with a greater abundance of keywords and an elevated level of importance attributed to the clusters (11). After clustering the keywords using the software, a total of nine clusters were identified. The details and covered keywords of each cluster are presented in Table 3.

**Table 3:** Results of the keyword cluster analysis.

Clusters	Size	Silhouette	Mean (year)	Top terms (LLR)
#0 Fear of falling	58	0.521	2016	Postural balance, older, prevention, elderly patients
#1 Machine learning	45	0.618	2017	Accidental falls, prediction model, natural language, processing, adverse drug event
#2 Older people	34	0.72	2015	Poly-pharmacy, patient safety, withdrawal, inappropriate prescribing
#3 Vision	33	0.723	2017	Potentially inappropriate, medication, visual impairment, cognition, performance
#4 Burden	30	0.661	2015	Injury, elderly people, fall assessment sheet, intervention
#5 Fall risk	29	0.719	2018	Elderly falls, total knee arthroplasty, fall risk, assessment, vestibular disorders
#6 Fall incident	29	0.699	2016	Disability, elderly patient, benzodiazepine receptor agonist, senile asthenia
#7 Parkinson’s disease	23	0.807	2017	Vestibular dysfunction, postural control, balance disorder, chronic disease
#8 Prescription	8	0.909	2017	Elderly women, complication, data reuse, vitamin D

### Analysis of keywords with strongest citation bursts

**Figure 7:**
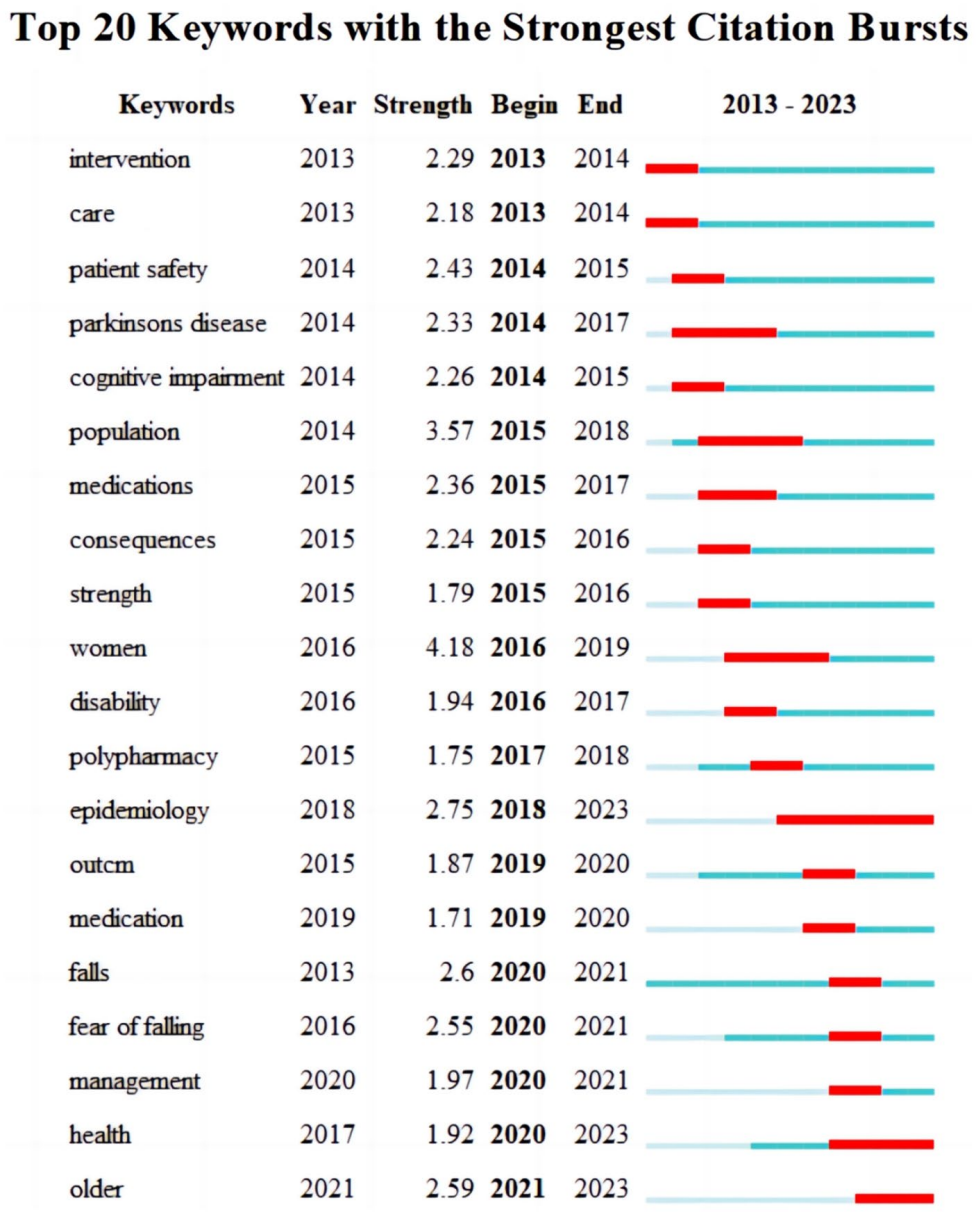
Top 20 keywords with the strongest citation bursts.

Identifying keywords with the strongest citation bursts is a widely utilized analytical approach in bibliometrics for determining the most commonly appearing keywords within a given body of literature. This method enables the identification of significant concepts and themes within a particular field of study, thereby helping researchers gain deeper insights into research trends, tendencies, and prominent issues within the discipline (12). As depicted in Figure 7, the top 20 keywords exhibiting the most significant citation bursts offer a more intuitive visualization of the shifts in research focal points and trends. Notably, research pertaining to epidemiology, aging, and health has attracted considerable scholarly interest since 2018 and may emerge as a prospective research trajectory within the field of falls among older hospitalized patients. Consequently, heightened attention should be directed toward this area with corresponding research endeavors.

## Discussion

Through visualization analysis of the fall research field in older inpatients within the Web of Science database from 2013 to 2023, we can draw the following perspectives. Over the past decade, there has been a shift in hotspots within fall research for older inpatients, transitioning from an initial focus on risk factors, prevention and treatment methods toward an increasing emphasis on the assessment and prediction of falls, postfall assessment and intervention, and treatment and rehabilitation of fall sequelae in older patients. Consequently, there is a noticeable trend toward diversification the study of falls in hospitalized older patients.

### Current status of research related to falls in older hospitalized patients

Falls in older hospitalized patients represent a significant medical and social concern due to their association with injuries, prolonged hospital stays, poor prognosis, and diminished quality of life. This issue has garnered widespread attention from scholars and clinical practitioners worldwide, leading to rapid development in research within this field. A review of the literature revealed that research in this area can be classified into four distinct categories:

#### Risk factors for falls

Currently, some researchers are utilizing risk factors as a point for conducting studies aimed at evaluating the likelihood of falls and establishing a foundation for subsequent interventions. A study conducted by Toshimitsu et al. demonstrated that visual impairment serves as an independent risk factor for falls in hospitalized patients (13). A study conducted by Amirabas et al. revealed that balance and gait impairments are significant risk factors for falls in older patients with Parkinson’s disease, necessitating the implementation of targeted interventions (14). A regional cross-sectional survey conducted by Zeliha et al. also demonstrated that older adults with impaired balance were more susceptible to falls (15). Haruki et al. demonstrated that the use of psychotropic medications was associated with an elevated risk of falls among older patients during hospitalization (16). A study conducted by Gu revealed that the utilization of potentially inappropriate medications may lead to a heightened incidence of falls among older patients during hospitalization (17). A study conducted by Helen demonstrated that central nervous system disease was correlated with a heightened risk of injurious falls (18). In conclusion, risk factors for falls in hospitalized older patients predominantly include visual impairment, balance and gait disturbances, medication usage, and central nervous system disorders, among other contributing factors.

#### Interventions to prevent fall

Fall prevention interventions have been shown to effectively delay the onset of falls in older adults. A study conducted by Amirabas demonstrated that vestibular rehabilitation training can enhance the gait and balance of older patients with Parkinson’s disease, thereby reducing the incidence of falls. (14)Gu and Mohammad demonstrated that evaluating the appropriateness of medication utilization in older hospitalized individuals can mitigate falls resulting from adverse drug reactions through effective drug management (17, 19). Wu and Singh proposed that enhancing the ward environment may lead to a decrease in falls among older hospitalized patients, offering a managerial perspective on reducing the incidence of falls (20, 21). Researchers have suggested a range of strategies to mitigate the risk of falls among older patients during hospitalization, including physical exercise regimens, medication oversight, and environmental adaptations.

#### The use of emerging technologies

In recent years, with the continuous advancement of science and technology, certain contemporary technologies have been utilized for investigating falls in older hospitalized patients. Alharbi’s study developed a monitoring and prediction system that integrates artificial intelligence to rapidly forecast the safety of hospitalized older patients and assess the risk of falls (22). Baig employed a wearable device to continuously monitor and record motion data, in conjunction with the patient’s vital signs, for the purpose of evaluating fall risk and predicting fall occurrence (23). Ioune designed an off-bed monitoring system utilizing sensor technology to mitigate the risk of falls (24). The utilization of artificial intelligence, sensor technology, and other advanced technologies is more systematic and intuitive than that of conventional methods. The incorporation of medical and scientific technology through emerging technologies facilitates the timely and efficient monitoring of older inpatients to prevent falls.

#### Rehabilitation and health management after falls

Spanò’s study demonstrated that both sequential (mixed) and simultaneous (dual-task) motor-cognitive training are effective interventions for improving walking balance, gait, walking speed, and fear of falling in older hospitalized patients with chronic cerebrovascular diseases, ultimately reducing the risk of recurrent falls (25). Health promotion through professional rehabilitation and scientific health management is a crucial approach to enhancing patients’ well-being. Therefore, investigating rehabilitation and management programs following falls represents a pivotal area of research in this field.

### Keyword co-occurrence and keyword cluster analysis

The co-occurrence analysis of keywords indicates that the centrality of “older adults,” “risk factors,” “older patients,” “meta-analysis,” “hip fracture,” and “falls” exceeds 0.1, signifying their significant positions in research over the past decade. To further explore these findings, we have chosen to focus on “risk factors” and “falls” as case studies for in-depth discussion. The co-occurrence analysis reveals a frequent association between these two keywords, suggesting a particular research interest in identifying and evaluating relevant risk factors related to falls. For instance, a study involving elderly hospitalized patients demonstrated an association between the use of hypnotic drugs was significantly associated with fall incidents (16). This underscores the importance of considering drug safety when developing preventive measures.

By conducting analysis of keyword cluster, we can further refine these clusters to pinpoint more specific research areas. For instance, the cluster related to “fear of falling,” with keywords such as postural balance and prevention, indicates a specific research focus on balance ability and preventive measures for the elderly. The “machine learning” cluster, which encompasses accidental falls and prediction models, signifies the application of machine learning technology in predicting fall events. Keyword co-occurrence not only reveals concentrated research topics but also interrelated scientific concepts while reflecting current directions focused on fall-related researches among elderly patients. Through this refinement process researchers are better equipped to precisely identify research questions, design targeted studies, and develop effective intervention strategies.

### Research hotspots and trends

The keywords exhibiting the most pronounced citation bursts signify the focal points and cutting-edge areas that have garnered significant scholarly attention within the field over time (26). As depicted in Figure 7, the evolution and progression of research hotspots and frontiers in the domain of falls among older hospitalized patients are obviously discernible over time. During the period of 2013–2014, intervention emerged as a prominent focus of investigation in this field, with a majority of researchers employing diverse interventions aimed at mitigating the incidence of falls. For example, Ooijen implemented the C-Mill gait-adapted treadmill-training program to modify gait patterns, thereby enhancing walking proficiency and consequently reducing susceptibility to falls (27). During the period of 2015–2018, there was a shift in the research focus within the field toward particular populations, with a particular emphasis on older hospitalized patients. For instance, Ikutomo conducted a study examining posttotal hip arthroplasty falls among patients in Japan (28). A study conducted by Patrick investigated the prognostic significance of a fall assessment instrument in the context of hospice care (29).

Since 2018, there has been continuous advancement in the field, leading to an increase in interdisciplinary collaborative research integrating epidemiology. For example, inquiries into fall rates (30), the development and validation of prediction models for falls (31), and the geographical distribution of falls (15, 32). The figure depicting the keyword with the most significant citation bursts reveals a sustained interest in epidemiology, aging, and health research since 2018. This trend is likely to continue into the future, making it a prominent area of study and a hotspot for researchers. Scholars in related fields should remain attentive to conducting relevant research in these areas.

### Future direction of researches

Based on the visualization analysis of existing literature and the limitations of current research, this study proposes potential direction for future research on falls among elderly inpatients. Firstly, there will be an expansion of interdisciplinary research to encompass additional disciplines such as epidemiology, physical therapy, and rehabilitation, with the aim of fostering enhanced cooperation and communication across disciplines. Second, in the realm of treatment and rehabilitation, future research is likely to concentrate on personalized health management and rehabilitation, as well as the development of tailored rehabilitation programs designed to align with the individual characteristics of older patients, ultimately leading to improved rehabilitation outcomes and overall health. With the advancement of artificial intelligence technology and the availability of big data, the utilization of intelligent medical technology in investigating falls among older hospitalized patients is poised to assume growing significance. This includes the implementation of monitoring systems and predictive models that integrate artificial intelligence.

### Limitations

In addition, the limitations of this study must be acknowledged. This paper offers only a macro view of trends rather than evidence direction and quality. Due to the limitation of CiteSpace software, only some databases (such as Web of Science) can be visually analyzed, potentially resulting in an incomplete analysis and the overlooking of significant research findings. Secondly, this study did not include peer-reviewed conference papers and reviews, which may not provide a more comprehensive macroscopic view of ongoing research in this area. Additionally, the exclusion of non-English literature may result in selection bias. This study only used CiteSpace software to conduct a visual analysis based on bibliometric methods, which may not fully capture the most important research hotspots, trends, and main knowledge trajectories. And it is worth noting that some bibliometric researches combined science mapping analysis with the main path analysis to reveal the development status and possible research fronts of the research domain from a dynamic perspective (33, 34). Therefore, in future researches, we can explore the potential application of these bibliometric techniques within fall among older hospitalized patients domain to gain profound insights and make groundbreaking discoveries. Only through the comprehensive application of multiple analytical tools and methods can we accurately and comprehensively grasp the progress and trends in research, thereby providing more compelling references for fall-related studies in older hospitalized patients.

## Conclusion

Our study offers a comprehensive macroscopic perspective on the trends in falls research among older hospitalized patients from 2013 to 2023. For instance, future research could integrate artificial intelligence and machine learning models to discern risk factors and assist healthcare professionals in formulating individualized preventive strategies. And interdisciplinary collaboration has the potential to foster synergy across diverse fields, thereby enhance the overall effectiveness of fall management. However, it is worth noting that healthcare professionals still need to use the “6S” evidence pyramid model for evidence retrieval and develop evidence-based fall management strategies. This study may help as a starting point for developing measures it may not be the main reference. Subsequent studies should delve deeper into these trends by assessing the quality and trajectory of evidence, with the aim of devising effective and personalized fall prevention and intervention strategies for elderly patients.

## Data Availability

The original contributions presented in the study are included in the article/supplementary material; further inquiries can be directed to the corresponding author.
